# Building alternative payment models in health care

**DOI:** 10.3389/frhs.2024.1235913

**Published:** 2024-06-14

**Authors:** Steven W. Howard, Natalie Bradford, Rhonda Belue, Margaret Henning, Zhengmin Qian, Kees Ahaus, Thomas Reindersma

**Affiliations:** ^1^ Health Services Administration, University of Alabama at Birmingham, Birmingham, AL, United States; ^2^ Public Health, University of Texas at San Antonio, San Antonio, TX, United States; ^3^ Health Sciences, Keene State College, Keene, NH, United States; ^4^ Epidemiology & Biostatistics, Saint Louis University, St. Louis, MO, United States; ^5^ Health Services Management & Organization, Erasmus University Rotterdam, Rotterdam, Netherlands

**Keywords:** alternative payment model, value based care, quality improvement, care coordination and care management, health care cost and utilization, health outcomes, health care innovation, health care transform

## Abstract

**Introduction:**

Global interest is growing in new value-based models of financing, delivering, and paying for health care services that could produce higher-quality and lower cost outcomes for patients and for society. However, research indicates evidence gaps in knowledge related to alternative payment models (APMs) in early experimentation phases or those contracted between private insurers and their health care provider-partners. The aim of this research was to understand and update the literature related to learning how industry experts design and implement APMs, including specific elements of their models and their choice of stakeholders to be involved in the design and contractual details.

**Methods:**

A literature review was conducted to guide the research focus and to select themes. The sample was selected using snowball sampling to identify subject matter experts (SMEs). Researchers conducted 16 semi-structured interviews with SMEs in the US, the Netherlands, and Germany in September and October 2021. Interviews were transcribed and using Braun and Clarke's six-phase approach to thematic analysis, researchers independently read, reviewed, and coded participants' responses related to APM design and implementation and subsequently reviewed each other's codes and themes for consistency.

**Results:**

Participants represented diverse perspectives of the payer, provider, consulting, and government areas of the health care sector. We found design considerations had five overarching themes: (1) population and scope of care and services, (2) benchmarking, metrics, data, and technology; (3) finance, APM type, risk adjustment, incentives, and influencing provider behavior, (4) provider partnerships and the role of physicians, and (5) leadership and regulatory issues.

**Discussion:**

This study confirmed several of the core components of APM model designs and implementations found in the literature and brought insights on additional aspects not previously emphasized, particularly the role of physicians (especially in leadership) and practice transformation/care processes necessary for providers to thrive under APM models. Importantly, researchers found significant concerns relevant for policymakers about regulations relating to health data sharing, rigid price-setting, and inter-organizational data communication that greatly inhibit the ability to experiment with APMs and those models’ abilities to succeed long-term.

## Introduction

There is increasing global interest in developing new value-based models of health care finance, delivery, and payment that could produce higher-quality, lower-cost outcomes for patients and society and reduce costs for payers. Alternative payment models (APMs) are aimed at incentivizing providers for improving patient outcomes, promoting more efficient care, and reducing unnecessary utilization (1–3). In doing so, APMs aim to move away from traditional activity-based payments such as fee-for-service (FFS), towards FFS linked to quality and value, APMs built on FFS architecture, and, ultimately, population-based payment (4).

The impact of government-sponsored APMs such as Medicare Accountable Care Organizations (ACOs) in the US is well documented (5), but significantly less is known about models in early experimentation phases or privately contracted between insurers and their health care provider partners. Further, considerations in designing and implementing APMs have received less attention than outcomes generated by such models (6). To gain a better understanding of the challenges providers and payers are faced with whilst designing and implementing APMs, we provide a synthesis of experiences and perspectives of industry experts across three countries that are forerunners in APM innovation: the US, the Netherlands, and to a lesser extent, Germany (7).

Valuable context is provided by the experts' experiences, perceptions, and beliefs regarding early-phase APMs across three countries. This enables us to enrich previous literature, shedding light on challenges of implementing APMs in multi-payer systems (8) and markets with possibly competing APMs (9). This study consequently offers insights into important design considerations concerning population and scope of care and services; benchmarking, metrics, data, and technology; finance, APM type, risk adjustment, incentives, and provider behavior; provider partnerships and the role of physicians; and leadership and regulatory issues.

## Methods

### Research design

This study adopts an exploratory qualitative approach to gain a comprehensive understanding of the design and implementation of early-phase and proprietary APMs, meaning APM models that are not detailed in the literature or public media. This would include APM models in private contracts between payers and provider organizations (very common in the USA), as well as models that will be shared publicly, but as of yet are too new for evaluation studies to be published. This research design allowed for in-depth exploration of the subject matter, where valuable context is provided by the participants' experiences, perceptions, and beliefs regarding early-phase APMs.

### Sample

In addition to reviewing the published literature, we began inquiry of our industry contacts in the US, including the St. Louis Area Business Health Coalition (BHC), and our professional networks in the US and the Netherlands. Beginning with academic partners and health care industry contacts, the research team used snowball sampling to identify subject matter experts working for insurance companies, provider organizations, and consulting firms in the US, the Netherlands, and Germany. The sample included: two US consultants with previous experience in Medicare ACO APM development; one German consulting executive experienced in establishing ACO-like APMs; two Dutch insurance firm executives with experience developing nationally-adopted APMs; one Dutch provider-organization executive experienced in developing a multi-provider-organization/network APM; one Dutch physician executive at an academic medical center; one physician leader of a large Dutch primary care organization with experience piloting new APMs; two Dutch health care administrators with experience launching APMs in hospital service lines; one senior leader at the Dutch National Institute for Public Health and the Environment; three US physician executives experienced in implementing APMs with insurers, government, and large employers; one senior manager of a German sickness fund (i.e., an insurer) with extensive experience in APM design, implementation and evaluation; and one German executive of a leading, value-based care-focused oncology clinic. The majority of participants were male (75%) and Dutch (50%). Over half of participants worked at provider organizations, 19% worked for insurers, 19% worked at consulting firms, and 6% worked in higher education or government. Most participants had multiple degrees. For example, nearly half of participants held PhDs, 63% had an MBA, MPH, Economics or similar master's degree, and half had clinical degrees (e.g., nursing, physiotherapy, medical or pharmacy). The majority held multiple degrees, so percentages do not sum to 100%. Table 1 presents the participants’ characteristics in detail.

**Table 1:** Participant characteristics.

	*N* (%)
Gender	
Female	4 (25)
Male	12 (75)
Country	
Germany	3 (19)
Netherlands	8 (50)
USA	5 (31)
Organization type	
Consulting	3 (19)
Payer/Insurer	3 (19)
Provider	9 (56)
Other/Governmental	1 (6)
Education type^a^	
Nurse, Physiotherapist, or similar	2 (13)
Medical Doctor	5 (31)
Pharmacist	1 (6)
Health Economist	3 (19)
MPH, MBA, MHA, or similar	7 (44)
PhD	7 (44)

^a^ Education types are not mutually exclusive, and do not sum to 100%.

### Data collection

The researchers conducted 16 semi-structured interviews with subject matter experts (SMEs) in the US, the Netherlands, and Germany in September and October 2021. Academic partners and SMEs who were not included as interviewees were consulted on instrument design, and their input shaped the questions for the semi-structured interview guide. Participants were contacted by email for interview appointments. Interviews took place at participants' offices, at a university partner's conference room, or by Zoom. All interviews were video recorded. The interviews were approximately 60–90 min and focused on the following questions developed through reviewing literature and consulting the SMEs: what were the key components of designing and implementing your APM? And who needs to be consulted or involved in design & implementation? The first question focuses on design and implementation of APMs, while the second question focuses on stakeholder involvement in developing APMs. The analysis presented in this study focuses on those two questions. However, additional questions were posed during the interview and are presented in Supplementary Appendix 1. In addition, we collected demographic data directly from participants and obtained professional profiles, including job title, highest degree achieved, organization name and organization type, from online sources.

Interview recordings were transcribed by two graduate research assistants and reviewed for accuracy by the interviewer and principal investigator. The process was facilitated by video and transcription app Descript (10). Interview data were pseudonymized after transcribing, and respondents consented to reuse the data for educational purposes (some respondents further consented to share video recordings of their interviews, and these can be made available upon request to the corresponding author).

The research design, including recruitment statement, interview instrument, and media release form, were approved by the Institutional Review Board of Saint Louis University. No honorarium was provided, and each participant provided informed consent before the interview.

### Analysis

We analyzed the transcripts using thematic analysis following Braun and Clarke's (11) six phase approach to thematic analysis. The six phases include: (1) becoming familiar with the data, (2) generating initial codes, (3) searching for themes, (4) reviewing potential themes, (5) defining and naming themes, and (6) producing the report. Six overarching themes and 16 codes were identified from the thematic analysis. The final themes are presented in Table 2.

**Table 2:** Themes, code categories, and interpretation.

Themes	Coding categories	Interpretation
1-Population & Scope of Care and Services	Population covered	Give a sharp definition of a substantial population
	Scope of services	Clarify goods and services that are included in the APM
2-Benchmarking, Metrics, Data & Technology	Benchmarking performance	Negotiate benchmarks in the payment arrangement
	Metrics	Use a carefully selected small palette of costs metrics, clinical metrics and patient-reported outcomes and experiences that can be directly influenced
	Technology & data analysis	Enable data sharing, support with technology and data analysis-expertise and provide meaningful data
3-Finance, Payment Model, Risk Adjustment, Incentives, & Influencing Provider Behavior	Financing sources	Consider the health system prevalent in the country
	Legacy systems	Bear in mind existing different payment systems for primary care, hospital care, rehab/elderly care
	APM type	Shift away from the wrong incentives, such as volume-driven payment. Include inflation and risk adjustment agreements
	Rewarding clinicians	Give providers freedom to decide whats best for their patients and reimburse for improvement
	Incentive transmission	Distribute payer savings due to prevention or good performance to groups and individual clinicians
	Multi-payer system	Consider how to deal within multi-payer systems with differences in contractual agreements
4-Provider Partnerships and the Physician's Role	Provider network issues (including hospital, clinic, labs, allied health services, etc.)	Assemble networks with physicians in the lead
	Role of the physician	Work on motivating agreements to change physician's practices to deliver value
6-Leadership and Regulatory Issues	Leadership	Physician leadership is essential in transitioning toward value-driven models
	Regulatory issues	Overcome the challenges of legal and regulatory issues (e.g., data, privacy, and anti-competitive regulations)

To reduce potential bias, two authors independently read, reviewed, and coded participants' responses related to the APM *Design and Implementation* question. The two authors subsequently reviewed each other's codes and themes for consistency, to assure that codes were not anecdotal or did not originate from a single code and to determine if themes could be merged into a higher order theme. Two of the other authors repeated this process to analyze responses related to the *Stakeholder Involvement* question.

To further reduce bias, one author who initially focused on interview responses related to the *Design and Implementation* question corroborated the codes and themes generated from the responses related to the *Stakeholder Involvement* question. One author in turn did likewise for *Design and Implementation* codes and themes. Discrepancies in coding were resolved by consensus.

## Results

### Population & scope of care and services

#### Population covered

Broadly recognized as the starting point for developing any APM, most participants discussed the populations to be served by their models and the scope of health care services to be covered. Populations served by the APMs varied widely from Medicare beneficiaries in the USA to pregnant women in the Netherlands. In addition to considering the patient population to be covered, participants explained that developing an APM also requires consideration of the number of patients to be served. Multiple participants mentioned the importance of having a large number of patients to justify the time and financial investment in developing an APM. Only when APMs cover a critical number of patients, desired effects can be realized, as one participant from the Netherlands mentioned:

*“And that is one of our key learnings, is that if you want to do alternative payment, you really need to have a substantial group of patients directly identifiable by a health care provider to intervene. If the group is too small, you don't get the change you want.”*

The size of the population is also determined by considerations pertaining to adequate spreading of financial risk. One participant recommended a minimum number of patients, stating:

*"I think that we recommended a minimum of 5,000 patients. So, if there*’*s a patient who spends a lot, then this will average out. And 5,000 (patients) is quite a considerable amount.”*

#### Scope

The patient populations were not the only features of the APMs that varied. Interviews revealed the scope of services covered under the APM arrangement also varied among organizations and countries. In most cases, all health care goods and services included under an overall financing scheme (e.g., Health Insurance Act in The Netherlands) were included in the APM, and what is kept separate in the financing scheme (e.g., prescription drugs) was not included. This was referred to as the APM covering the total cost of care for a specific population. Other APMs focused on patient populations in a certain setting. For instance, one participant described an APM covering inpatient hospital care for stroke patients, subsequent inpatient rehabilitation care, and related services by physicians and physiotherapists, where in-home care and post-discharge care are excluded.

Related to determining the scope of care and services that should be included in an APM, participants spoke about the physician's ability to impact utilization of goods and services—the ability to “steer” patients. To effectively do so, it is important to understand where utilization—but also spending—happens, leading to APM arrangements having specific scopes:

*“And within the Medicare Accountable Care Organization space, it really is understanding where that spending and utilization is happening.”*

### Benchmarking, metrics, data, and technology

#### Benchmarking and metrics

Not all but multiple participants discussed how benchmarking performance measures is critically important for successful APMs. The need to negotiate performance benchmarks early in the process of creating an APM was a persistent theme across interviews. Participants explained it is critical for the payer and providers to set benchmarks including those impacting how cost savings are determined and distributed. As one participant noted:

*“What percent of the savings stays with the payer, what percent goes to the provider? Likewise, if the end point after adjusting for risk and inflation is above the benchmark, there is loss, and you negotiate in the contract ahead of time. Do we agree that some of the loss gets moved from the [insurance] plan to the provider? How much of the loss? Is there a limit on the loss?”*

Benchmarking was based on a national comparison or on the organizations' own performance history. In the US, participants remarked that they were bound by national benchmarks imposed by a federal agency. In one Dutch primary care bundled payment model, a participant said the foundation of the benchmark for spending was “*a 3-year weighted average of the GP's historical spending multiplied by a national growth rate.”* Another participant from the Netherlands explained how when their organization began experimenting with APMs, they were more concerned with their own organization's improvement, and did not use national benchmarks:

*“We were not yet interested in the national benchmark; we just want to see if the hospital has improved compared to the last few years in this hospital.”*

Multiple insurers may impose multiple benchmarks, presenting organizations with the question of which metrics to use and strive for in their benchmarks. One participant stated that the benchmark metrics that are most difficult to achieve are those they strive for the entire population.

Not all services and associated spending may be readily controllable by the providers contracted for a given APM. Cost metrics included in benchmarking may be only those addressable or directly influenced by the clinicians in the APM, or they could be for the total cost of care (TCOC). To address this issue, some benchmarks were adjusted to compare only populations similar to the physician's or clinic's patient panel or population. Interestingly, one initiative includes all the patients in the catchment area of the provider in the benchmark, whether treated or not by said provider.

*“So, if […] you have one hospital and a very complex patient will be treated in another region, that could be done by the hospital to lower the risks of complication and increase the chance of savings. And that is why we include it in the contract of that hospital to say: even if you didn't treat that patient, it was in your catchment area, we still take it into account for your savings and your quality measures.”*

With respect to catchment area benchmarking, such adjustments help correct for differences in demographic variables like age, ethnicity, immigration status, education or income levels that can impact health literacy, care-seeking behavior, health service utilization, and total cost of care. Benchmarks were also adjusted for health care cost inflation, to eliminate patient cases that were spending outliers and to eliminate cherry-picking within the provider's catchment area. The data on which benchmarking is based differs across settings, with data coming from providers or insurer's internal data or national databases and registries of cost, utilization, and outcome data.

In addition to the need for metrics for benchmarking, participants talked about what metrics should be used. Metrics on outcomes, process and cost were used separately or in combination, for instance through the use of scorecards. Participants also alluded to the importance of striking a balance between using process and outcome metrics. Physicians rely on process metrics rather than outcome metrics during cycles of care, and are thus necessary for stimulating certain provider behavior:

*"If you do the process correctly, you're more likely to have a better outcome, which is what physicians rely on along the way.”*

In addition to the range of metrics, participants also discussed the number of metrics used and the importance of keeping the amount manageable:

*“To put 50 metrics out in front of a provider to perform on, that takes away from their primary focus. So, if we can get them to focus on the most important ones, the other stuff should fall in line with it.”*

#### Data and technology

Core barriers preventing more health care organizations from adopting APMs are the lack of congruency between data and clinical practice, the dearth of meaningful data, and the absence of technology and analysis expertise to mine data and convert it into information that physicians can act on.

For instance, participants discussed the issue of enabling physicians to ensure they understand the APM and how to be successful under it. Providers need training in the processes and technological tools to help them better achieve metrics, and they need frequent benchmarking feedback so they can understand their own performance compared to their own history and to peers. For example, in comments about the need to support providers, participants mentioned paying more attention to sharing *“some aggregated data sets”,* the need for “*in-person support and coaching on efficiency,”* and making data “*accessible in the workflow”*.

Providers face the challenge of having data but being limited in their ability to make use of it. One participant spoke specifically about the lack of data transparency explaining that “*we collect a lot of data, but we don't share it.”* Even those physicians or organizations that can analyze their own data are unlikely to have access to their peers' or partners' data, which is important if the APM payer is holding legally separate but partnered organizations to collective cost and quality performance targets. This may be caused by legal hurdles, but there can also be delays as centralized data repository organizations compile, deidentify and structure reports on the data for providers to view and exchange. Participants who reported the greatest success with data sharing and patient management tended to be sharing a common electronic medical record (EMR) or one that could interface with other EMRs.

Beyond data sharing, participants were concerned physicians might lack the data analysis and technological expertise necessary to implement APMs. For example, a participant stated:

*"We deemed not every GP practice capable of participating in the Shared Savings Program. [Some providers] had their own analysts so they could really analyze what was going on and where they could improve. [Many] don't really have the capabilities to understand the statistical techniques that we use. For example, they are not capable of performing benchmark analyses themselves.”*

However, larger health systems indicate that they do have the skills and resources, with dedicated data analytics teams keeping track of performance and informing physicians and management. These systems are poised to conduct much more sophisticated analysis, including using artificial intelligence and machine learning to better understand their collective data and predict which patients are most at risk or have “care gaps” that need to be targeted, and which treatments are most likely to succeed.

### Finance and payment model, risk adjustment, incentives, and influencing provider behavior

#### Sources of financing

The sources of financing for the APMs discussed by the participants were varied and tied to one or more of the major health insurance models prevalent in each country. In some cases, including Germany and the Netherlands, part of the funds received by provider organizations from insurers or government may include special financing for novel policy initiatives, including APMs or other quality improvement endeavors, as well as the efforts to evaluate them. One participant explained the challenges of starting a new APM initiative:

*"So, we got about, I think 4 million euros from [the payer] for the first years, and 1 million was earmarked for evaluation. But today it is very difficult for sickness funds, in a more competitive environment to push money in before the savings arise. So, our contracts today are in the way that we invest ourselves into these projects for the first years and see until the end of the second year, what happened in the first year. So, we have a long time-gap between knowing what the costs of care are and what the savings are.”*

#### Legacy systems

In all the APMs participants described, the financing was paid to insurers by government, enrollees, or both. The subsequent step—from payer to provider—is where payments begin to vary. In general, APMs are bound to and undergirded by legacy systems such as FFS. In Germany, insurers must pay providers FFS for all government-approved services on the price list, whether they are individual providers or provider organizations. For hospitals, these FFS payments took the form of diagnosis-related group-based payments (DRGs), and physician payments were weighted on points-based schemes, similar to the resource-based relative value scale (RBRVS) FFS system used in the US and elsewhere. For example, one participant from Germany explained:

*"We have different remuneration systems. We have a kind of fee for service point scale in the outpatient sector. We have the DRG in the inpatient sector. There was an attempt in [our insurer] three years ago, I'm not sure about the results, but we talked about the “hybrid DRG”: the same services provided in the inpatient or outpatient sector get the same payment to overcome this gap between the two sectors.”*

Consistently, interviewees mentioned complications with a multi-provider payment system due to diversity in payment structures between different health care entities. For example, one interviewee mentioned that*:*

*“In the Netherlands, so it*’*s very complicated. Hospitals are paid for service, but it*’*s another payment system for hospitals than for rehabilitation.”*

Interviewees revealed that in the Netherlands and US, insurers, providers, and provider groups are permitted to freely negotiate payment arrangements. In Germany however, payers and APM experimenters reported having little-to-no ability to share savings or pay providers for anything not on the fee list, which has to do with clinging onto legacy systems previously described.

#### APM type

Within the boundaries set by legacy systems, there is a wide variety of payment models, each targeted at different levels and with or without metric-driven financial incentives to change provider behavior. One participant in Germany discussed obtaining the approval of special FFS payment codes to pay physicians for the care coordination and care management. Beyond the direct payment for medical services, it was less common for insurers to offer providers or provider groups financial incentives for improving costs and quality. However, sometimes, the payments included a component to help fund providers' innovations in learning what approaches can improve outcomes and experiences. One participant explained about their clinic organization being involved in a capitation scheme that includes funds for the clinic to invest in new quality and outcome initiatives:

*“We came to a general basic fee, which they [the insurer] pay us for every patient. And no matter if we don't see that [patient], or if we see the patient 100 times, that*’*s what they pay us.”*

One participant reflected on moving from volume-based FFS to incentive structures that refocus on reducing waste and unnecessary utilization, and increasing pursuit of prevention, maximizing patients' health, lower cost treatment alternatives, and adherence to evidence-based practices:

*“Another example could be that you would have to follow up on patients that are unnecessary just because you need to write your bills for the consultations, which is something we don't need to do anymore. If it*’*s unnecessary, we don't ask patients to come back.”*

It has long been recognized that paying uniform capitation levels to insurers or providers can incentivize risk selection (seeking out only low-risk patients). To address this risk and incentivize the enrollment and care management of high-risk patients (like those with multiple chronic diseases), payers (government and private payers) have various mechanisms in place to adjust capitation or similar payments upward for each higher-risk patient, or for a patient population. Risk selection and risk adjustment issues are well understood in the health care payer and provider community, but implementing risk adjustment is sometimes avoided because of its complexity (e.g., accounting for case-mix in APMs).

#### Rewarding clinicians

We found most APMs providing well-defined, metric-driven financial provider organizations to achieve goals. Now, as more data become available on outcomes and patient experiences, at least a portion of the financial incentive is increasingly transitioned from process metrics to outcome metrics and patient experiences.

*"I think the core of accountable health care is not that we say to a care provider, okay, you have to do this, and you have to do this, and then we give you savings. It*’*s a bit strange if you say, I have a reward for you, but you get this reward or maybe you have to pay a penalty, and at the same time, I'm going to tell you exactly what you need to do. That*’*s not fair, right? So basically, what we say is, okay, we are going to give you a shared savings payment, or you have to pay the loss, but you decide yourself what you think is best for the patient. The core of accountable care is that you give providers the freedom to decide themselves what is best for the patients.”*

Although steering and incentivizing on outcomes is increasingly preferred, it remains important to keep emphasizing process metrics as well, because it forms important steering information for clinicians during care cycles.

Not all financial incentives are metric-driven. For instance, a shared savings agreement described by a participant stimulates prevention because GPs are encouraged to look for patients in an early stage of cardiovascular disease, preventing escalating costs for which the GP is held accountable.

*“The basic idea is that we hold our GP financially accountable for health care spending, not only in his own practice, but also outside of his practice.”*

#### Incentive transmission

Participants specified how incentives were driven down to the individual provider level, with one interviewee explaining that at the end of the year:

*“[…] every Medicare Advantage plan gives a kicker [bonus] to the provider for an annual wellness visit [physical check-up] because they think that*’*s the visit that'll lead to improved risk scoring and diagnosis capture.”* [authors' explanatory note: In the U.S. Medicare Advantage system, the participating private insurers (“Medicare Advantage plans”) are incentivized to enroll individuals with complex health needs. This is accomplished using risk-adjustment based on documenting patients' health conditions (including chronic conditions like diabetes, heart disease, etc). More annually-documented conditions will yield greater capitated revenue from the U.S. federal Medicare program.]

In addition to the possible preventive health effect, this subsequently brings greater risk-adjusted revenue into the insurer. Similarly, another participant described how savings are allocated.

*"[…] part of the savings come back from the insurer and you [the provider] can use these funds to reinvest in your practice.”*

An important precondition for distributing savings is that the organization has to perform according to the contract terms in order to receive a share of the savings in the first place, which can then be parsed out to the individual providers or physician groups. Groups can then compensate their clinicians at their own discretion. Physician compensation may be tied to how well they perform on certain aspects.

#### Multi-payer systems

There are several considerations that are inherent to working in multi-payer systems, where providers have contracts with multiple payers. This leads to dilemmas of what practice model to adopt and addresses issues of spill-over:

*“If I have a value-based contract […] and I'm going to transform and do the things that I need to do to be successful there. But how do we deal with the other 70%? Do I have two different models that feel incredibly difficult? Do I deliver the same intensity and level of care to the 70% under fee for service when I know I won't be reimbursed at the same level?”*

In line with this, it is difficult for providers to switch between two or more practice models from one patient to the next, which, perhaps unrealistically, also requires knowing which patient is under which model. Describing this issue, a participant shared:

*"The new problem is differential treatment for patients in the ACO vs. patients that are not in. The doctor is saying: half of my patients can be supported in a different way than the regular way, and the regular way is still the default version. So, we are looking for a situation that we might have, let*’*s say 70% or 80% of the insurers, so that the new default version is the integrated care model, and the old one, the regular one is only for 20% of patients, and even then, it*’*s the way that these get the services as well.”*

Another challenge during the transition from legacy FFS payment models to APMs is the rate at which the provider's patient panel moves over to the new model.

Considering other payer aspects, a participant in the US stated the belief that innovation in APMs in the US is slowing, as the largest insurers already have models with which they are comfortable, and lack interest in further experimentation, or novel APM suggestions from their provider partners:

*“It*’*s an ACO-like total cost of care model paying physicians and provider groups FFS against a target-budget. [Insurers] are not creating these models anew; they're iterating on existing models… Every year they make updates to their total cost of care ACO deals. Now they require downside risk and the like. It*’*s much harder. You have to have more members and there*’*s other bars you have to clear to get into those deals.”*

### Provider partnerships and the physician's role

#### Provider network issues

This theme examines how physicians and allied providers play a pivotal role in any APM. How those providers are contracted and involved in the APM arrangements varies. However, in our interviews, participants discussed the approach and issues in assembling, developing, and maintaining provider networks (including hospital, clinic, labs, and allied health services).

Assembling networks involves a critical evaluation of providers' readiness to become part of the network. This process includes “stage-gating” providers based on their maturity level and experience with risk-based payment, with corresponding adjustments to the incentive and payment models that align with their growth. Another participant articulated the flexibility afforded to providers in joining or leaving networks, explaining that providers “may decide to go with the new system, or they stay with the old system.” This participant went on to explain the importance of creating value for multiple types of providers in their network:

*“We have to look for win-win solutions. What is the win for a physical therapist to work with us, or a pharmacist? Producing health, because he might fear that he loses some kind of activities that he could otherwise get reimbursed [under the legacy FFS model].”*

Other participants described more difficulty in assembling networks of hospitals, rehab centers, physiotherapists, community-based nurses, and other caregivers, prompted in part also by the different financing and payment regimens under which they operate.

#### Role of the physicians

The participants were consistent in emphasizing the essential leadership role and core influential role of physicians, especially PCPs and GPs.

*“[Our model is] sort of the Physician-as-Team-Captain model with medical assistants, physiotherapists, occupational therapists, pharmacists, counselors, etc. on the team.”*

Supporting physicians in adopting new practice styles is an important topic. Physicians hence need:

*“[…] practice transformation to be able to succeed in these types of arrangements. They need human in-person support and coaching on efficiency. Simply showing docs their metrics is not enough. They need workflow changes to be proactive. You bake the data into the workflows to allow docs at point of service to improve patients’ health.”*

Ensuring the establishment of processes and essential components such as accurate patient data, enables effective physician performance and resource allocation to support practitioners. Another important enabler of physician performance is that payment models should provide a level of security from the onset, allowing a gradual transition to risk-based working (e.g., pay-for-performance):

*“So, I think, if you're starting to get into the space, the pay-for-performance model gives a little bit of that security, so you're not diving right into risk and writing checks at the end of the performance year.”*

### Leadership and regulatory issues

#### Leadership

Strong leadership is essential for success in designing and implementing APMs, particularly because they require collaboration between disparate groups of health care providers and payers, each with its own business and financial interests, and each with its own challenges in transforming away from the legacy FFS models of health care payment and toward APMs. While the Dutch interviewees were not explicit about leadership, it was clear from the interviews that strong leadership required service to the providers and seeking consensus as much as is feasible. One of the German participants had the most to say about the issue of leadership in the context of innovating new APMs:

*“Leaders need to be bold in experimenting. The cultural norm in Germany [for insurers and providers] is to simply stick with the ‘normal train’. Trying new things is risky, both financially and for a leader*’*s career.”*

Besides physician leadership being of utmost importance, as already illustrated before, director-level leadership must not be forgotten because of the amount of financial and organizational resources that need to be mobilized. In short, transitioning toward APMs needs to be bottom-up and top-guided.

#### Regulatory issues

In all three countries represented by interviews in this study, legal and regulatory issues were pervasive challenges, and most participants shared extensive thoughts on this topic. This is also the case for communicating costs to implement APMs:

*"We know the laws, sometimes that*’*s the slowest thing to change. We have a ‘watchdog’ [regulator] who guards the payment systems. It*’*s on a national level, and you cannot communicate your costs or declarations to other health care organizations. So that*’*s very complicated if you work together with one bundle. If you don't know what hospital costs are and if you don't know what rehabilitation costs are, you may not talk about it, then it*’*s very difficult to work within one bundle.”*

In the US, the federal agency Centers for Medicare and Medicaid Services extensively regulates the Medicare and Medicare Advantage programs, where most of the early APM innovation has taken place. As recognized thus far, care coordination and data sharing are essential functions for better integrating health care delivery, measuring performance against metrics, and sharing funds under an APM. Additionally, electronic medical records, patient privacy, and anti-competitive regulations can impede or facilitate the success of APM implementations. In the Netherlands and Germany, interviewees talked about the challenges that European General Data Protection Regulation (GDPR) patient privacy regulations have caused:

*“The GP has to keep it secret. In our version of the GDPR—because countries are allowed to give their own interpretation—the things insurers can do with these data are kind of listed exhaustively. So, you really have to have a good reason why you're sharing this individual data. For us it*’*s a bit tricky because if we make a mistake, we get a fine. So yeah, I think this is one of the biggest barriers for us.”*

In Germany, the regulatory environment was even more challenging than in the Netherlands. In addition to GDPR restrictions on data sharing, German regulations historically also required any changes to fees or provider-payer interaction rules be agreed to by all insurers serving a given geographic service area. Participants explained how this made experimentation, like APMs, difficult.

*“Prior to 2000, the law required full consensus by all insurers in an area to all adopt any changes. After 2000, that the law allowed for development of contracts between single sickness funds (insurers) and groups of providers or management companies who have contracts with groups of providers. … So, in the first system… there was a special paragraph in the law that allowed insurers to push money into a system like this [an APM] and then retract the money from the normal providers on the other regions. So, they don*’*t pay it, but they had some surplus money for pushing it into the new project. That helped us for the startup.”*

However, there seems to be increasing understanding by regulators to diminish regulatory barriers to APM implementation. These findings further indicate that regulatory issues differ per country, and issues materialize on the local as well as national level.

## Discussion

To better understand APM design model considerations, particularly related to experimental and private, yet-unpublished models, we interviewed subject matter experts in the Netherlands, Germany, and the US. Participants represented diverse perspectives of the payer, provider, consulting, and government areas of the health care sector. We found design considerations had the following five overarching themes: (1) population and scope of care and services, (2) benchmarking, metrics, data, and technology; (3) finance, APM type, risk adjustment, incentives, and influencing provider behavior, (4) provider partnerships and the role of physicians, and (5) leadership and regulatory issues.

Overall, the results were aligned with previous literature. Hayen et al. (12) proposed building blocks for shared savings APMs that included many of our themes, particularly the first three themes. However, their research focused intently on the contractual details of the shared savings arrangement, including clearly defining populations and services, accounting for risk, benchmarking and metrics, details on calculating savings, and algorithms for sharing it (12). Importantly, we found insights on additional aspects not previously emphasized, particularly the role of physicians in leadership, practice transformation, care processes, patient health behaviors, and health promotion necessary for patients and their providers to thrive under APMs. Furthermore, the European Union's EIT Health knowledge network published a 2020 report on value-based health care in Europe (13). While VBHC is a broader concept than APM (APMs are common subsets of VBHC), we observed overlap between the themes identified in our study and the report. While our participants identified the importance of clearly defining the patient population in question, the report did not cite the need to focus on specific medical conditions. Further, participants in our study provided insights into the realities and challenges of the legal and regulatory environments that hampered their abilities to develop and promote wider adoption of APMs in their specific countries.

Another finding relates to the four broad categories of APMs identified by the Health Care Payment Learning and Action Network (4). Models described by our participants were most consistent with APMs with shared savings and downside risk (category 3). However, instances of FFS linked with quality (category 2) and population-based payments (category 4), such as the Dutch primary care clinics and the stroke patient model were also observed. Our findings suggest that this categorization could be the result of APM maturation, as providers gradually mature from category 2 to 4, with getting used to working with risk-based contracts and getting their data infrastructure ready. This emphasizes that providers may need more time to learn to work with risk-based contracts (14). Assuming that systems are still learning to adapt to APMs and given the importance of provider involvement in the APM contracts, the lack of payer interest in involving them in further innovation in APMs that we signaled in our findings could bode poorly for improving long-term health outcomes and could lower provider enthusiasm for continued partnership in APMs.

Our results also offer additional perspectives on the populations covered by APMs. Previous research has shown how payment reform has spillover effects on the populations not targeted by APMs (15, 16), and our results indeed show hesitancy on the part of providers to roll out practice transformations associated with APMs to only a subset of their patient populations. Two spillover mechanisms were identified: through a high-level approach of rolling out the practice transformation across all patients after a majority (or certain percentage) of patients is under any kind of APM contract. The other mechanism pertains to performance metrics, which differed between payers' APMs and of which providers often committed to the most stringent performance targets for the entire population.

Publications by Porter and colleagues emphasized the theoretically optimal design of APMs, with preference for bundled payment models (1, 2, 17). Their recommendations were consistent with this study's participants' emphasis on metrics and benchmarking, though they focused more on individual patient-level measures, and not population-level (or physician panel-level) measures. Like the participants here, these previous studies also promoted the use of PROMs and PREMs but recognized that current limitations of data availability impact the ability to create robust outcome measures.

Concerning benchmarking and metrics, increasing emphasis is placed on outcomes vs. processes, stimulating providers to attain desired outcomes without prescribing *how* to attain them (18). However, our results show that organizations still find it important to retain process measures, as they provide short-cycle feedback for physicians during episodes of care. Our findings further seem to indicate that applying a parsimonious set of performance metrics to the provider's entire patient population is preferred, which corresponds with previous calls for aligned metrics (19).

Our findings underline that disparate providers have different IT systems, differing abilities to capture data, and varied capabilities for sophisticated data analysis, all of which complicate the data sharing that is essential for coordinating patient care across multiple, independent payer and provider organizations. We observed significant concerns relevant for policymakers about regulations relating to rigid governmental price-setting and billing code restrictions, health data sharing, and inter-organizational data communication that inhibit the ability to experiment with APMs, that pose challenges to those models' abilities to succeed long-term.

### Limitations

There are a number of limitations to this study. Chiefly, the small, non-random sample of participants across a small number of health care organizations in only three countries, may limit the generalizations. Second, the snowball sampling might introduce selection bias. However, previous literature has identified these three countries are forerunners in APM development (7). Additionally, the body of scholarly and trade literature is regularly authored and read by the same subset of health care researchers and practitioners. Seeking input from a niche group of experts in this way can introduce bias, to the extent they read about the same new value-based care and APM approaches, and share collective views (20). Although the number of interviews was small, interviews were in-depth, and saturation was reached among the participants. It is important to underline that the aim of this study was exploratory, and researchers sought to better understand the experiments and unpublished private APMs emerging in multiple countries.

### Conclusion & implications

This work provides insight into the major issues organizations consider in designing and implementing their APMs. An impactful quote that captures an overarching finding and emphasizes the implications of this study's findings for practitioners is one participant's admonishment that “*it's not enough to simply have the contract in place, it's so much more: partnerships, tech, practice transformation, aligned incentives, training, removing barriers like time-conflicts and disincentives, conflicts of interest.”* This statement underscores the importance of moving beyond the usual contractual terms and focus on learning and relational aspects of APMs. To improve the chances for APM success, it is important to integrally involve physicians in model design, transform care delivery practices to be more proactively and prevention-minded (and less volume-based), and for policymakers to remove unintended policy barriers. Payers need to make training and other resources available to providers, and to ensure they work together toward transforming the way physicians and other providers are practicing. For governments, this means removing, or establishing waiver processes for regulations that are impeding payers' and providers' efforts to transform health care and awarding starter grants to promising payment innovation projects.

Potential avenues for further research could explore the organizational readiness and maturity of providers, particularly in adapting to the challenges posed by risk-based contracts inherent in APMs. Further exploration into how payers and providers assemble networks and assess the appropriateness of providers to participate could yield valuable insights. In light of our findings emphasizing the importance of moving beyond contractual terms, additional research on relational governance within networks and between payers and providers would be valuable, especially given the growing focus on APMs in the health care landscape.

## Data Availability

The datasets presented in this article are not readily available because release of final transcripts and/or videos may still be pending final approval of participants. Requests to access the datasets should be directed to showard3@UAB.edu.
