# Supplementary material for: Building alternative payment models in health care
Source: Front Health Serv. 2024 Jun 14;4:1235913. doi: 10.3389/frhs.2024.1235913 (PMC11211624; doi:10.3389/frhs.2024.1235913)
Supplement: Supplementary file 1 [file Datasheet1.pdf]

SAINT LOUIS UNIVERSITY
EST. 1818

DEPARTMENT OF HEALTH MANAGEMENT AND POLICY

3545 Lafayette Avenue
St. Louis, MO 63104

P 314-977-8100
F 314-977-6310

www.slu.edu

July 9, 2021

SAINT LOUIS UNIVERSITY and
ERASMUS UNIVERSITY

Semi-Structured Questionnaire Guide (with probes)

The purpose of this project is to better understand the inspiration, organization, implementation, and evaluation of Value-based Health Care / Alternative Payment Models in healthcare organizations in the U.S. and Europe. By "Value-based Care" or "APM" we mean payment models that move away from paying providers for volume of care provided, and that aim to incentivize providers to emphasize quality & safety of care, improved patient outcomes, reducing unnecessary utilization, and promoting health.

Our interview should take approximately 1 hour. You may stay for as much of the interview as you feel comfortable, and may stop at any time. Our meeting will be video recorded, if you are comfortable (if the person is not, offer audio-only instead… s/he will have already known about the default video recording policy, but this is a reminder and confirmation).

Semi-Structured Interview Questions (probe for clarification as needed):

1. "How do YOU define Value-Based Care (&/or Alternative Pymt Model)? Describe your model" (or your most innovative / "flagship" model(s) if you have multiple). How long has it been in place (or where are you in the process now… may include % of patients, % of contracts, % of spend$$? Rev$% in VBC? More advanced stage & managing Total Cost of Care, or earlier-stage & on P4P-only?) Capture attributes like NFP/for-profit, IPA/health system-centered, etc.

2. "What were the problems or the background that led you to see the need for a VBC/APM model?" (details about their market dynamic & its influence… year-over-year cost increases? Payer pressure? CMS MACRA, QIP & other regulatory-driven pressures? Seeking new revenue sources?)

3. "What were the key components of designing your VBC/APM model?" What aspects of Care Delivery Transformation vs. Payment/Finance Model Reform? Provider concerns? Payer/Employer concerns? Fed/State Regulatory concerns? Business processes / Administrative streamlining needed? (ie; prior auths, etc) E.H.R.-enabled Quality Reporting? Or claims data? Evidence-based measures? Analytic abilities? Physician leadership? Specialist Bundles or ??

SAINT LOUIS UNIVERSITY
EST. 1818

DEPARTMENT OF HEALTH MANAGEMENT AND POLICY

3545 Lafayette Avenue
St. Louis, MO 63104

P 314-977-8100
F 314-977-6310

www.slu.edu

4. "Who needs to be consulted or involved in design & implementation, and necessary partners?" (Provider Advisory Groups? Employer Forums? Brokers? NCQA / agencies? Large Employers? Patients? Health System? Insurer? Administrators/leadership? Finance/Actuarial?)

5. "How would you evaluate the VBC/APM model? How has it worked?" (probe various kinds of outcomes: financial (Total Cost of Care? Or sub-components of Total Cost?), efficiency, health outcomes, quality metrics, patient satisfaction, staff/provider satisfaction, Star Ratings, etc)… impacts of Regulations? (any regs impeding your success?) Are measures 'handicapped' if starting at disadvantage? MA metrics: MER/MLR, Star Rating, RAF scores, Covered-lives growth… Medicare MSSP ACO: Quality rating, $ shared savings, RAF, Attributed lives… Commercial/Medicaid: MER/MLR, HEDIS/Quality, varied RAF algorithms…
All Payers: ED p1k (NYU method), Inpatient p1k (% ambul.-sensitive conditions, empaneled patients & growth, Chronic Dx-cohort utilization & costs…

6. "What is your advice for other organization interested in launching their own VBC/APM models? How can they have the best chances for success?"
Fully understand finc'l/econ. Model & how to achieve it?
Must have Physician & Admin. Leadership understanding/support/champions?
E.H.R. ability/workflows for ID'ing population & tracking quality measures.
Master ID'ing population assigned/attributed, & using E.H.R. & claims data.

Thank you so much for your time.
Reminder that you will get a copy of the final edited version of our interview.
No video will be released until you have had a chance to review it.
